# Macrophages and Iron: A Special Relationship

**DOI:** 10.3390/biomedicines9111585

**Published:** 2021-10-30

**Authors:** Stefania Recalcati, Gaetano Cairo

**Affiliations:** Department of Biomedical Sciences for Health, University of Milan, 20121 Milan, Italy; stefania.recalcati@unimi.it

**Keywords:** macrophage, iron, ferroptosis

## Abstract

Macrophages perform a variety of different biological functions and are known for their essential role in the immune response. In this context, a principal function is phagocytic clearance of pathogens, apoptotic and senescent cells. However, the major targets of homeostatic phagocytosis by macrophages are old/damaged red blood cells. As such, macrophages play a crucial role in iron trafficking, as they recycle the large quantity of iron obtained by hemoglobin degradation. They also seem particularly adapted to handle and store amounts of iron that would be toxic to other cell types. Here, we examine the specific and peculiar iron metabolism of macrophages.

## 1. Introduction

Macrophages, differentiated cells of the mononuclear phagocyte system, are essential for the innate immune surveillance and the induction of the inflammatory response. They also have a variety of other key homeostatic functions as resident cells in several tissues [1]. Despite their large heterogeneity, a common feature of macrophages (i.e., big eating cells) is their scavenging capacity, as they are particularly able to phagocytose pathogens, apoptotic cells and cellular debris. The major targets of homeostatic phagocytosis by macrophages are old/damaged red blood cells (RBC). Erythrophagocytosis is a demanding task for macrophages: since 200 billion RBC are produced each day, an equivalent number must be removed by reticuloendothelial macrophages to maintain a balanced red cell mass and avoid both anemia and erythrocytosis. By doing so, macrophages return hemoglobin-derived iron to the circulation, thereby providing most of the iron to meet the requirement of erythropoietic cells, which acquire almost exclusively transferrin-bound iron and use it for hemoglobin synthesis and cell proliferation [2]. These iron recycling phagocytes are macrophages resident in the spleen red pulp and liver (Kupffer cells), which depend on the activity of the heme-responsive Spi-C transcription factor for development and acquisition of this specialized function [3].

Ferroportin is the only known protein that exports ferrous iron from the cytoplasm across the plasma membrane and is key for the iron-releasing activity of macrophages (reviewed in [4,5]). Thereafter, the ferroxidase activity of circulating ceruloplasmin allows the conversion of iron to the ferric form that is bound by transferrin, which delivers this essential metal to all the cells of the body that use iron in a variety of enzymatic reactions. Since excess iron can be toxic, a strict balance of iron levels must be maintained [6]. At the systemic level, iron homeostasis is mainly regulated by hepcidin, a liver-derived protein transcriptionally induced by plasma and tissue iron through the BMP-SMAD signaling pathway [7]. Hepcidin induces the internalization [8] and the E3 ubiquitin ligase RNF217-mediated [9] degradation of iron-loaded ferroportin in cells involved in iron transport, thus operating as a negative feedback regulator of iron recycled from macrophages as well as intestinal absorption in duodenal entrocytes [10].

In this review, we describe the most recent and relevant insights into the molecular aspects of macrophage iron trafficking with some reference to non-infectious pathophysiological conditions, in line with the aim of this Special Issue.

## 2. Iron Handling by Macrophages

### 2.1. Uptake

**Figure 1:**
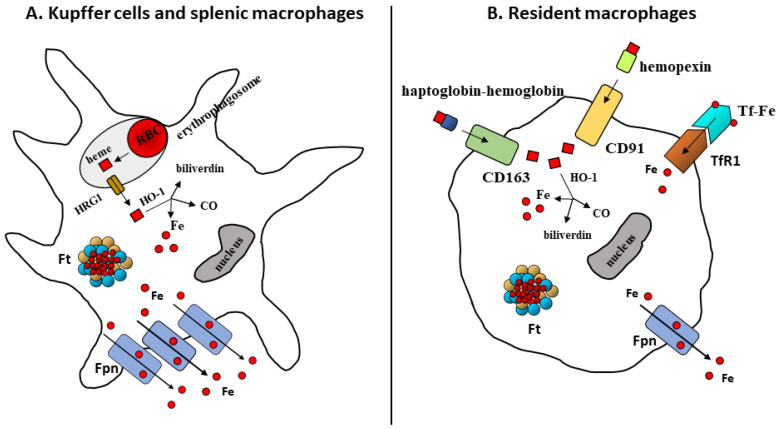
Iron trafficking in macrophages. (**A**) Macrophages specialized in erythrophagocytosis, for example in the spleen and liver (Kupffer cells), mainly acquire heme iron derived from the ingestion and degradation of red blood cells (RBC). Following erythrocyte internalization, heme derived from hemoglobin and exported from phagolysosomes to the cytosol by HRG1 is catabolized by heme oxygenase (HO-1); the resulting iron is mainly exported by ferroportin (Fpn) or stored in ferritin (Ft). (**B**) Other types of macrophages can obtain iron through several alternative mechanisms. These resident macrophages process a lower amount of iron and acquire both heme iron through the LRP1/CD9 and CD163 receptors, which scavenge hemopexin and haptoglobin, respectively, and transferrin-bound iron via transferrin receptors (TfR1).

Macrophages in the red pulp of the spleen and the Kupffer cells in the liver, which recognize cell surface alterations and hence capture and internalize RBC with a process similar to classic phagocytosis, mainly acquire heme iron. Ingested erythrocytes are engulfed in phagolysosomes, in which hemoglobin and heme are degraded by oxidants and hydrolytic enzymes (Figure 1). Heme is cleaved in the endoplasmic reticulum by the inducible form of heme oxygenase 1 (HO-1), yielding CO, iron and biliverdin. Despite the presence of iron transporters such as natural resistance-associated macrophage protein (Nramp1) at the erythrophagosomal membrane, a recent study showed the key role of heme transporter HRG1 (Heme-responsive gene 1). This protein, which is highly expressed in macrophages and specifically localizes to the phagolysosomal membranes, transports heme from the phagolysosome and is essential for heme-iron recycling in macrophages [11]. The transport of heme across the phagocytic vacuole membrane to the cytoplasm, where it is degraded by HO-1, is in line with the topology of HO-1, which is tethered to the endoplasmic reticulum with the catalytic domain facing the cytosol. It is possible that part of cytoplasmic heme is exported from macrophages by feline leukemia virus, subgroup C, receptor 1a (FLVCR1a) [12], but it is relevant to note that the pathway relying on HO-1 activity is essential at both local and systemic levels. In fact, it has been shown that the absence of HO-1 is lethal for liver and spleen macrophages and is accompanied by defects of iron homeostasis and kidney damage in mice [13] and humans, though the phenotype of the single human patient lacking HO-1 was milder than in mice [14].

Since an average macrophage ingests at least one red cell/day [15], erythrophagocytosis represents the major mechanism of iron uptake; however, macrophages can use several different routes to obtain iron (Figure 1). It is possible that these alternative pathways play a more important role in the variety of macrophages that do not have erythrophagocytosis as their main function. Though monocytes express a low number of transferrin receptor (TfR1) molecules, the majority of tissue-resident macrophages, which are embryonically settled in organs where they are maintained by self-renewal [16,17,18], express TfR1. The acquired transferrin-bound iron is then reduced by six-transmembrane epithelial antigen of the prostate 3 (Steap3) and transported in the cytoplasm by the divalent metal transporter 1 (DMT1) [19]. Moreover, macrophages can also internalize heme iron by means of scavenger receptors, which can represent an important source of iron under conditions of hemolysis or tissue damage. In fact, to prevent the release of potentially toxic free heme, cell-free hemoglobin is bound by haptoglobin, and the resulting complex is then bound by the macrophage transmembrane receptor CD163. In case free heme forms, an additional protection is offered by hemopexin, which scavenges heme and is then recognized by low-density lipoprotein (LDL) receptor-related protein 1 (LRP1/CD9) [20,21]. Given the lipophilic nature of heme, there is also the possibility that it is acquired by macrophages without the activity of heme-delivering transporters, possibly through hydrophobic channels (discussed by [22]). However, these emerging pathways and the possible effects on macrophages in hemolytic conditions are still not well understood.

### 2.2. Storage

Cytosolic iron that is not used by phagocytic cells to synthesize proteins involved in vital functions or exported by ferroportin is deposited in ferritin, an iron storage protein composed of H and L subunits, which is induced by heme and iron [23]. Ferritin synthesis is mainly controlled at the post-transcriptional level by the activity of iron regulatory proteins (IRPs), which maintain cellular iron balance by binding to conserved elements in target transcripts, which also include mRNAs coding for ferroportin and TfR1 [24]. IRP binding activity, which prevents ferritin mRNA translation, is decreased when cellular iron levels are high, thereby allowing efficient ferritin synthesis. Conversely, under conditions of iron scarcity, elevated IRP activity inhibits ferritin synthesis and iron sequestration. However, ferritin expression can be stimulated by iron also at the transcriptional level [25]. Moreover, in order to keep cellular iron availability under control, nuclear factor erythroid 2-related factor 2 (NRF2), a transcription factor responsive to heme that induces HO-1 [26], also triggers the transcription of both H ferritin [27] and ferroportin [28]. Ferritin content is also determined by nuclear receptor coactivator 4 (NCOA4), which stimulates ferritin degradation (ferritinophagy). NCOA4 specifically binds ferritin and delivers it to lysosomes where it is degraded, thereby increasing iron availability [29]. This mechanism, which is controlled by iron and oxygen levels through hypoxia inducible factors [30], may be beneficial in macrophages where it is accompanied by iron release, as it would sustain erythropoiesis, particularly under conditions of iron deficiency [31].

In mammals, the two types of ferritin subunits co-assemble in various H:L ratios forming heteropolymeric 24-mer proteins, called iso-ferritins, with tissue-specific distribution [23]. For example, H-rich ferritins having low iron content are found in tissues with high ferroxidation activity, such as the heart (~90% H, 10% L) and brain, while those containing L-rich iso-ferritins, which accumulate larger amounts of iron, are present in tissues having a storage function, such as the spleen (~10% H, 90% L) and liver (~50% H, 50% L). In line with the iron exchange activity of macrophages, it has been reported that ferritin in monocytes is H-rich: analysis of ferritin subunit composition showed that L/H ratio was 0.5 in blood mononuclear cells [32]. In the J774A.1 mouse macrophage cell line, the H subunit was almost three times more abundant than the L counterpart [33].

The key role of H ferritin in macrophages has been underlined by the impaired iron storage capacities caused by H ferritin deletion in the myeloid lineage, which was accompanied by higher iron levels and faster iron turnover, eventually favoring the growth of intracellular pathogens [34,35].

### 2.3. Release

The only regulated mechanism that is known to export iron from cells depends on the activity of ferroportin. This protein, whose expression is regulated at several levels [36], transports ferrous iron from the cytoplasm across the plasma membrane and is highly expressed in macrophages (reviewed in [4,5]). Notably, the transcription factor Spi-C, which allows macrophage differentiation toward the iron recycling phenotype [3], activates ferroportin expression [37], thereby underscoring that ferroportin-dependent iron export is a key feature of this macrophage population. Consequently, under physiological circumstances, the macrophage displays little stainable iron because most of the iron is rapidly released. Pioneering studies of plasma iron kinetics in human volunteers established the presence of two pools: a fast-releasing pool, which promptly returns iron derived from hemoglobin to circulation within 30 min, and a slow-releasing pool based on iron stores that exports the remaining iron over the course of days. Interestingly, the amount of iron released in these two distinct phases was influenced by erythroid demand and altered under pathological conditions such as inflammation and hemochromatosis, which mainly affected the fast-releasing pool in opposite directions [38].

We can now interpret these events on the basis of the interaction of hepcidin with ferroportin. In fact, inappropriately low hepcidin levels in hemochromatosis patients accounts for the observation that macrophages are paradoxically iron-deficient despite body iron overload [39]. Conversely, the increase in JAK-STAT signaling-dependent hepcidin synthesis triggered by cytokines (particularly IL-6) downregulates ferroportin activity, thereby inhibiting iron release under inflammatory conditions [4,5]. For still unexplained reasons, ferroportin-mediated iron release from macrophages seems more sensitive to hepcidin than intestinal export, as patients unable to synthesize active ferroportin accumulated iron in lamina propria macrophages but not in enterocytes [40], although the different life span of the two cell types may also play a role in these settings.

Increasing evidence suggests that ferroportin-mediated iron efflux from macrophages plays a relevant role not only to recycle iron to the circulation (as described above), but also to control iron availability in tissue microenvironments in both physiological and pathological settings (reviewed in [5]). For example, iron release from alveolar macrophages and skin resident macrophages is important for lung function and hair growth, respectively. Additionally, ferroportin expression by macrophages is required for optimal wound healing, and iron provided by local macrophages plays a key role in tumor growth and atherosclerotic plaque progression (reviewed in [5]). The essential role of ferroportin in iron export is highlighted by the embryonic lethality of ferroportin deletion in mice and by studies showing that it is required for systemic and local iron homeostasis (reviewed in [4,5]). However, it has been reported that iron can be transported across the endolysosomal membrane by the nonselective cation channel TRPML1 [41]. Yet, its role in macrophages has not been characterized, apart from preliminary data showing that both TRPML1 and TRPML2 are downregulated by iron in the macrophage cell line THP1 [42].

Alternatively, iron could exit from macrophages through ferritin. Studies aimed at characterizing the source of serum iron showed that ferritin is mainly secreted from macrophages. In fact, splenectomy strongly reduced the increase in circulating ferritin observed in mice with IRP2-targeted deletion [43]. In addition, spleen and liver macrophages lacking ferroportin expression were iron loaded and accompanied by increased serum ferritin [44]. Therefore, ferritin secretion may represent a complementary mechanism for iron release to the bloodstream or the tissue microenvironment, though it should be noted that serum ferritin is iron poor [23].

Lipocalin-2 (Lcn-2), a carrier protein belonging to the lipocalin superfamily with high affinity for bacterial siderophores, may represent an additional protein involved in iron export from macrophages. The role of Lcn-2 as an iron exporter has been mainly characterized in tumor-associated macrophages. Apoptotic tumor cells induced the polarization of macrophages toward the alternative M2 phenotype and stimulated expression of iron-bound Lcn-2 [45]. Notably, it has been shown that Lcn-2 was sufficient to provide iron for breast cancer progression [46].

### 2.4. Subcellular Distribution

Once it reaches the cytosol, iron has to be delivered to various subcellular sites (e.g., the mitochondria). The molecular control of the partitioning between storage, release, incorporation into nuclear iron centers, and use in the assembly of heme or iron–sulfur clusters depends on chaperon proteins: the poly(C) binding proteins, PCBP1 and PCBP2. By playing a key role in both the transport and cytosolic distribution of iron for storage in ferritin, and utilization for a variety of biochemical reactions or ferroportin-dependent efflux [47], these proteins may be involved in the control of the long-known biphasic nature of iron release from macrophages, which has been described by the elegant ferrokinetic experiments reported above.

It should be noted that in order to play a large variety of distinct functions, macrophage populations are able to acquire different phenotypes (polarization), comprising two major groups: pro-inflammatory (M1) macrophages and alternative anti-inflammatory (M2) macrophages [1]. We have demonstrated that, in addition to a different behaviour and gene expression profile [48], polarized macrophages also show a distinct expression of genes involved in iron homeostasis [49,50]. In particular, thanks to high ferritin expression, M1 cells withhold iron, whereas M2 are prone to ferroportin-mediated iron release. Iron sequestration in M1 macrophages is thought to be mainly a bacteriostatic mechanism for host defense, acting at both the systemic (hypoferremia) and local levels to restrict iron access to extracellular microbes [49,51]. This strategy, however, may eventually lead to the development of anemia in the setting of chronic inflammatory diseases [52]. On the other hand, the iron export activity of M2 macrophages is probably functional to the growth of adjacent cells in the microenvironment. This Janus-like function may favor efficient tissue repair [53], but also the growth of tumor cells [5,46]. The differences in iron metabolism existing in the various macrophage subsets can be relevant also for immunometabolism, as iron may be involved in the regulation of energy production and amino acid catabolism [54].

## 3. The Iron Resistance of Macrophages

Through several mechanisms, macrophages generate oxidants, which play a key role in their effector functions to eliminate pathogens. Nevertheless, pro-inflammatory macrophages are able to survive for several days in a stressful environment rich in highly reactive molecules. The macrophage antioxidant response is key to protecting mitochondria and other organelles, proteins, and nucleic acids from oxidative damage. The pentose phosphate pathway flux that generates NADPH is particularly active in macrophages and represents an important cofactor in the production of both oxidants, such as superoxide anion during the respiratory burst, and antioxidants, such as glutathione (GSH) and thioredoxin, which limit oxidative damage. A metabolic shift to the pentose phosphate pathway has been shown to confer protection also to heme loading in a mouse model of sickle cell disease [55]. In addition, recent reports indicate that the transcription of antioxidant genes through the NRF2 pathway represents an important cytoprotective strategy (reviewed by [56]). Moreover, macrophages use vitamins (E and C) and other antioxidant molecules, such as GSH, to resist the oxidative burden. These phenomena are particularly evident in pro-inflammatory M1 macrophages and more limited in alternatively activated M2 macrophages [57], which produce less oxidant species and are involved in tissue repair (reviewed in [58]). In this context, since the catalytic action of iron is key for the transformation of less reactive oxidants into highly toxic hydroxyl radicals [6], the iron content of macrophages is important for their resistance and viability.

Contrary to other cells, which acquire iron through TfR1-mediated diferric transferrin acquisition only when they need iron, macrophages process heme-derived iron, which is their major source of the metal. How can macrophages deal with such a large amount of iron without incurring cell damage and eventually ferroptosis, a form of regulated cell death in which, as implied by the name, iron plays a key role [59]? In ferroptosis, unrestrained formation of oxidant species triggered by increased levels of intracellular redox-active iron results in a lipid peroxidation process that cannot be counteracted by glutathione peroxidase 4 (Gpx4) antioxidant activity.

Indeed, it should be remembered that each RBC contains more than one billion atoms of iron, and it has been established that rat liver macrophages (Kupffer cells) can ingest and process up to one erythrocyte/hour [60], though on average each macrophage clears approximately one RBC per day. The amount of iron acquired by macrophages through erythrophagocytosis should be compared with the amount of iron that a cell can obtain by internalizing transferrin-bound iron. The values of TfR1 recycling kinetics strongly depend on experimental factors (cell type, growth conditions, etc.) and are influenced by iron availability [61,62], but it can be assumed that a cultured cell expresses on its surface approximately 1 × 10^5^ TfR1 molecules able to internalize diferric transferrin. It has been calculated that each receptor requires on average 15 min for a complete cycle; therefore, a standard cell can acquire by this pathway no more than 1 million iron atoms/hour. This means that it has to handle about one thousand times less iron than a spleen macrophage or Kupffer cell. While this situation of cellular iron overload is typical of iron recycling reticuloendothelial macrophages, which do not live in an environment rich in oxidants, we would like to remember that macrophages can internalize large amounts of heme iron also under pathological conditions that are characterized by an oxidative microenvironment, such as tissue repair [53] or atherosclerotic plaques [63].

**Figure 2:**
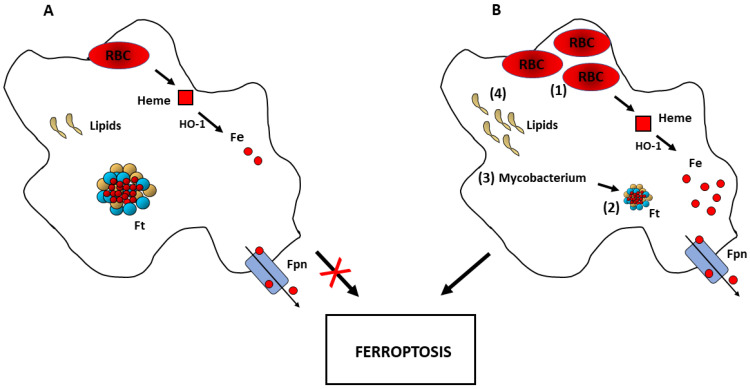
Macrophages and ferroptosis. (**A**) The low sensitivity of macrophages to ferroptosis under conditions of physiological RBC ingestion depends on efficient expression of HO-1, ferritin and ferroportin, which protect the cell from heme and iron load. (**B**) These homeostatic defense mechanisms are not sufficient to prevent ferroptosis in case of increased iron content caused by excessive erythrophagocytosis (1) or ferritin degradation (ferritinophagy) (2) triggered for example by Mycobacterium tuberculosis infection (3). Considering the role of polyunsaturated fatty acids in ferroptosis, an excess of lipids can also facilitate ferroptosis of iron loaded macrophages, for example in the context of atherosclerotic plaques (4).

Some types of cells of the innate immune system (e.g., macrophages and microglia) are not significantly prone to pro-ferroptotic stimulation [64]. In fact, physiological RBC ingestion and iron recycling do not seem to have any adverse effects on macrophage function. The results of a recent study in which iron accumulation in mouse macrophages caused by ferroportin deletion did not alter insulin resistance led the authors to conclude that macrophages have a remarkable capacity to tolerate iron excess [65]. In addition, in response to slightly enhanced erythrophagocytosis, macrophages respond by increasing their expression of HO-1, to degrade heme, and ferritin, to safely store the resulting free iron. This response allows the cell to handle the increased heme and iron load, thereby protecting it from severe oxidative injury (Figure 2). In line with these findings, it has been recently shown that specifically increasing H ferritin levels by repressing NCOA4 and inducing mitochondrial ferritin expression is important to modulate the sensitivity to ferroptosis of macrophages challenged with the ferroptosis inducer RSL-3 [66]. Conversely, the lack of HO-1 exacerbated the tumor necrosis factor α-dependent and oxidants-dependent damage and cytotoxicity in macrophages exposed to excessive heme [67].

Activated M1 macrophages, in comparison to their M2 counterpart, are less sensitive to pharmacologically induced ferroptosis, a difference which has been shown to depend on nitric oxide production [68]. A similar high resistance has been reported in mice in which ferroptosis was triggered by myeloid cells-targeted deletion of Gpx4 [69]. Notably, Gpx4 is dispensable for maintenance of lung, peritoneal and spleen macrophages at steady state, thereby underscoring the intrinsic resistance of these cells to ferroptosis. We speculate that different iron compartmentalization may also play a role in this context, as safe iron storage in ferritin, which is a feature of M1 macrophages [50], could confer higher resistance to ferroptosis.

As described above, another mechanism conferring resistance to iron burden is the capability of macrophages to rapidly get rid of excess iron, thanks to high ferroportin expression (Figure 2). Moreover, macrophages present multifaceted functions: phagocytosis with its activation of biochemical pathways is only one of the skills of macrophages, which are multifunctional depending on need and environment, and can change properties and phenotypes [70]. In fact, they are not only scavenger cells but also regulatory cells displaying active synthesis of mediators. Hence, not all the iron acquired by macrophages needs to be sequestered or exported, as it may be extensively used for a variety of enzymes involved in the production of mediators, which are heme or iron proteins (proteins of mitochondrial oxidative metabolism, peroxidases, cyclooxygenases and lipoxygenases).

However, these homeostatic mechanisms can be overwhelmed, thereby leading to cell death. In fact, in a mouse model of transfusion, after rapid ingestion of a large number of RBC the increase in HO-1 was insufficient to prevent ferroptotic cell death of red pulp macrophages triggered by substantial erythrophagocytosis [71].

In the same way, despite the high resistance of macrophages to heme iron shown by the lack of overt phenotype in mice with hemopexin and haptoglobin inactivation [20], hemolytic stress may increase the cellular burden of iron beyond the capacity of defense mechanisms, until macrophages become unable to cope by storing and/or releasing iron [20].

Iron excess does not necessarily have to come from an external source such as RBC, as shown by a study demonstrating that ferroptosis occurs in Mycobacterium tuberculosis-infected macrophages [72]. Since autophagy has been shown to promote ferroptosis through degradation of ferritin (ferritinophagy) [29,73], the autophagic process induced by Mycobacterium could serve as an inductive stimulus for intracellular iron release and ferroptotic macrophage death. Similarly, it has been demonstrated that ferritinophagy can lead to ferroptotic cell death in osteoclasts which, like macrophages, are cells of the myeloid lineage [74].

It seems, therefore, that macrophages, thanks to their resistance to iron overload, are less susceptible to ferroptosis, although they can eventually die in case of massive iron overload (Figure 2). However, imbalance of another factor involved in ferroptosis, such as impaired antioxidant capacity or lipid overload as occurring in atherosclerotic lesions (reviewed in this Special Issue by [75]), may trigger regulated cell death. In fact, it has been recently demonstrated that iron overload, which has no obvious effect on normal THP-1 macrophages, can induce ferroptosis in THP-1 cells exposed to oxidized LDL (a model of atherosclerotic foam cells) [76].

## 4. Conclusions

In the present review, we highlighted the molecular control of iron trafficking in macrophages, an expanding area of investigation that, in recent years, has provided additional advances in knowledge and understanding of the multiple functions of these versatile cells. A major aspect of the relationship between iron and macrophages is the key role of these cells in host defense and erythroblast development in the bone marrow; these functions have been recently reviewed, (see for example [5,77]). On the other hand, in line with the purpose of this Special Issue, which is aimed at the description of the involvement of macrophages in homeostasis and in the pathogenesis of non-infectious pathophysiological conditions, we covered iron trafficking in macrophages. In particular, we addressed the mechanisms that enable reticuloendothelial cells to withstand iron levels orders of magnitude higher than any other cell. In addition, macrophages are able to expand their storage capacity to accommodate additional iron under inflammatory conditions [49,70,77]. Notably, the apparent low toxicity of iron accumulated in the reticuloendothelial system following multiple transfusions, as compared to the serious damage that similar amounts of iron imparted to parenchymal organs in hereditary hemochromatosis, was already well-recognized by clinicians decades ago [78]. However, we also described specific conditions, such as hemolytic stress, which increase the burden of iron beyond the macrophage capacity to cope by exporting, storing or utilizing iron, eventually resulting in levels of intracellular iron that trigger ferroptosis. Sometimes, iron operates in connection with alterations of other metabolic processes involved in the cell’s susceptibility toward ferroptosis. Given the role of ferroptosis in a variety of diseases, new insights into the mechanisms underlying the resistance of macrophages to this form of regulated cell death are likely to improve our understanding of this process and generate interesting perspectives for its therapeutic modulation.

## Data Availability

Not applicable.
